# Early postoperative controlling nutritional status (CONUT) score is associated with complication III-V after hepatectomy in hepatocellular carcinoma: A retrospective cohort study of 1,334 patients

**DOI:** 10.1038/s41598-018-31714-w

**Published:** 2018-09-07

**Authors:** Lei Li, Chang Liu, Jiayin Yang, Hong Wu, Tianfu Wen, Wentao Wang, Bo Li, Lvnan Yan

**Affiliations:** Department of Liver Surgery & Liver transplantation center, West China Hospital of Sichuan University, Chengdu, 610041 China

## Abstract

Postoperative complication III-V is closely related with hepatectomy-related mortality for hepatocellular carcinoma (HCC) patients. The aim of the study was to investigate the relationship between CONUTS and postoperative complication III-V. 1334 HCC patients who underwent hepatectomy were divided into two groups: high CONUTS group (early postoperative CONUTS ≥ 8, n = 659) and low CONUTS group (early postoperative CONUTS < 8, n = 675). The characteristics and clinical outcomes were compared and analyzed. Risk factors for postoperative complication III-V were evaluated by univariate and multivariate analysis. early postoperative CONUTS showed a good prediction ability for postoperative complication III-V (AUROC = 0.653, *P* < 0.001), with the cut-off value of 8. The high CONUTS group had higher incidence of postoperative pulmonary complications (12.0% vs 7.9%, *P* = 0.011), bile leakage (2.6% vs 0.9%, *P* = 0.018), intra-abdominal hemorrhage (4.9% vs 1.6%, *P* = 0.001), postoperative liver failure Grade C (3.6% vs 1.0%, *P* = 0.002), complication III-V (15.6% vs 6.2%, *P* < 0.001), length of ICU stay > 48 hours (9.4% vs 4.1%, *P* < 0.001) and mortality in 90 days (2.6% vs 0.4%, *P* = 0.001), longer period of postoperative hospitalization (10 (8–13) vs 9 (7–11) days, *P* < 0.001). Multivariable analysis revealed that early postoperative CONUTS ≥ 8 (OR = 2.054, 95%CI = 1.371–3.078, *P* < 0.001) was independently associated with postoperative complication III-V. Early postoperative CONUTS ≥ 8 was identified as a novel risk factor for postoperative complication III-V, and should be further evaluated as a predictive marker for who are to undergo liver resection.

## Introduction

Liver cancer was the fourth leading cause of cancer death according to the Global Burden of Disease Study 2015^1^. The most common type of primary liver cancer is hepatocellular carcinoma (HCC), followed by cholangiocarcinoma^2^. Surgical partial hepatectomy is widely regarded as the preferred curative treatment for patients with HCC. Postoperative mortality and morbidity for HCC patients have been reduced significantly, however the recurrence rate in 5 years remains close to 70%^3^. Previous studies^4,5^ suggested that perioperative nutritional supplementation could reduce the postoperative complications and shorten the duration of hospitalization of patients who undergo liver resection for cancer. Hsieh CE *et al*.^6^ reported that postoperative nutritional support could reduce pulmonary complications, promote the recovery of liver function and shorten length of stay in adult liver donors. As we know, malnutrition is considered to be associated with worse outcome of critical illness and appropriate nutritional intervention can improve outcomes for inpatients. There has been no uniform definition of malnutrition, different nutritional evaluation methods were reported and widely used. However, few studies investigated the relationship between nutritional assessment scores and postoperative outcomes.

**Figure 1:**
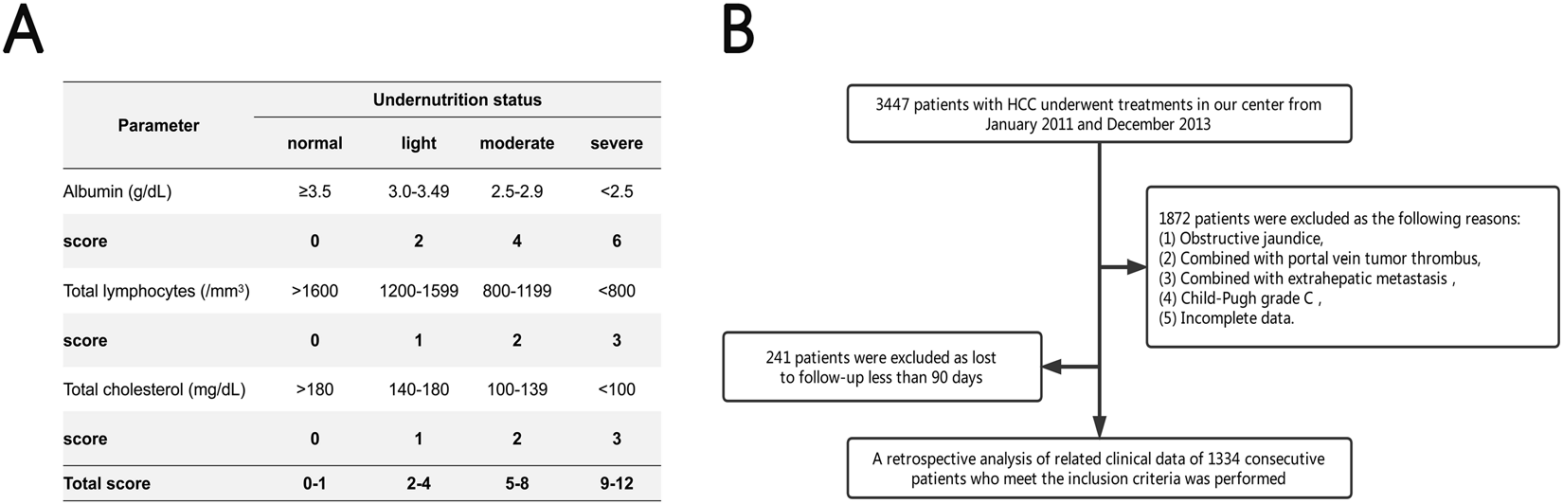
(**A**) Assessment of undernutrition status by the CONUT score. Ignacio de Ullibarri J. *et al*. Nutr Hosp^11^. (**B**) Flow diagram for 1334 consecutive patients who meet the inclusion criteria.

Several immune-nutritional factors were reported as potential predictor for the outcomes of HCC patients after hepatectomy, such as aspartate aminotransferase to platelet ratio index (APRI)^7^, fibrosis index based on the four factors index (FIB-4)^8^, albumin-bilirubin score (ALBI)^9^, prognostic nutritional index (PNI)^10^. The Controlling Nutritional Status score (CONUTS), first validated and reported by de Ulíbarri J, I. *et al*. in 2005, as a screening tool for early detection and continuous control of hospital undernutrition^11^. The formula, consists of serum albumin, cholesterol levels, and total lymphocyte count, is shown in Fig. 1A. Previous studies have been reported that preoperative CONUTS could predict the poor prognosis in patients who with colorectal cancer^12^, hepatocellular carcinoma^13^, or esophageal squamous cell carcinoma^14^. However, hemodynamics and blood constituents would be affected, due to intraoperative blood loss and fluid infusion. Hence, we hypothesized that early postoperative CONTS would be superior to preoperative CONUTS in predictive ability. A retrospective cohort study was carried out to investigate the relationship between early postoperative CONUTS and complication III-V after hepatectomy in HCC patients.

## Materials and Methods

### Study population

The study included 1334 consecutive HCC patients who underwent treatment at West China hospital of Sichuan university between January 2011 and December 2013. Hepatocellular carcinoma diagnosis was confirmed based on the current EASL^15^ or AASLD^16^ HCC management guidelines. The inclusion criteria followed: (1) Pathological diagnosis confirmed hepatocellular carcinoma, (2) received partial hepatectomy by open or laparoscopic hepatectomy, (3) patients >18 years. Exclusion criteria included the following: (1) patients with obstructive jaundice, (2) combined with portal vein tumor thrombus, (3) combined with extrahepatic metastasis, (4) liver function of Child-Pugh grade C, (5) loss to postoperative follow-up within 90 days, (6) poor data integrity. The flowchart was revealed in Fig. 1B. We collected the medical records containing the demographics, preoperative laboratory values, imaging examination data and postoperative clinical outcomes from the clinical liver cancer database of the department of Liver Surgery & Liver Transplantation Center, West China Hospital of Sichuan university.

### Perioperative management

Careful history analysis, physical examination and routine preoperative laboratory measurements were performed for all patients. Routine imaging examination to evaluate the tumor and cardiopulmonary function evaluation was carried out before surgery as we previously described^8^. Antiviral drugs were administered to patients with positive HBV-DNA before the operation, and postoperative continuous antiviral therapy follow the current guidelines^17^. Patients were operated under general anesthesia and intraoperative ultrasonography was used routinely. Hepatic vascular inflow occlusion (hemihepatic or total hepatic blocking) or the Pringle maneuver was utilized according to the surgeon’s preference in most patients as those previously described^18,19^. Hepatectomy was performed using the clamp crushing or hooking with ligation method, ultrasonic dissector with coagulator. Based on preoperative and intraoperative condition, patients were transferred to the intensive care unit for treatment when necessary.

### Parameter definition

Early postoperative CONUTS was calculated from the first postoperative serum albumin level, lymphocytes count and total cholesterol level, the blood samples were obtained within 12 hours after operation. Postoperative CONUTS change was calculated by subtracting the preoperative CONUTS from the early postoperative CONUTS. Clinically relevant portal hypertension (PHT) is defined as the presence of esophageal varices and/or a platelet count of less than 100 × 10^9^/L in association with splenomegaly^20^. The Clavien–Dindo complication classification system^21^ was used for postoperative complication grading and grade III-V complications were defined as severe complications. Postoperative liver failure^22^ (PLF) and biliary leakage^23^ were defined in accordance with the criterion of International Study Group of Liver Surgery. PLF was classified into three categories (grade A, B, and C)^22^. Liver resection with more than three segments was defined as major resection, or as minor resection. Mortality was defined as any death occurring from the time of surgery up to 90 days after hepatectomy.

### Statistical Analysis

Scientific research secretaries were trained to take the collection and analysis responsibilities. Continuous variables were reported as mean (standard deviation [SD]) or median (interquartile range [IQR]). Student t test for continuous variables with parametric distribution. Mann–Whitney U test or Kruskal–Wallis H test for those with nonparametric distribution. Categorical variables were reported as numbers and percentages, and compared using Pearson χ^2^ analyses or Fisher exact test. The predictive ability of potential factors for postoperative complication III-V was assessed by the corresponding area under the receiver operating characteristic (AUROC) curve. Youden index was utilized to choose the optimal cut-off value, which set as the value maximizing the sum of sensitivity and specificity. To identify risk factors for postoperative severe complications, all significant factors in the univariate analysis were used for multivariate analysis by the forward stepwise logistic regression. All statistical analyses were performed using IBM SPSS Statistics software 21.0, and statistical significance was set at *P* < 0.05, with two-tailed.

## Result

**Figure 2:**
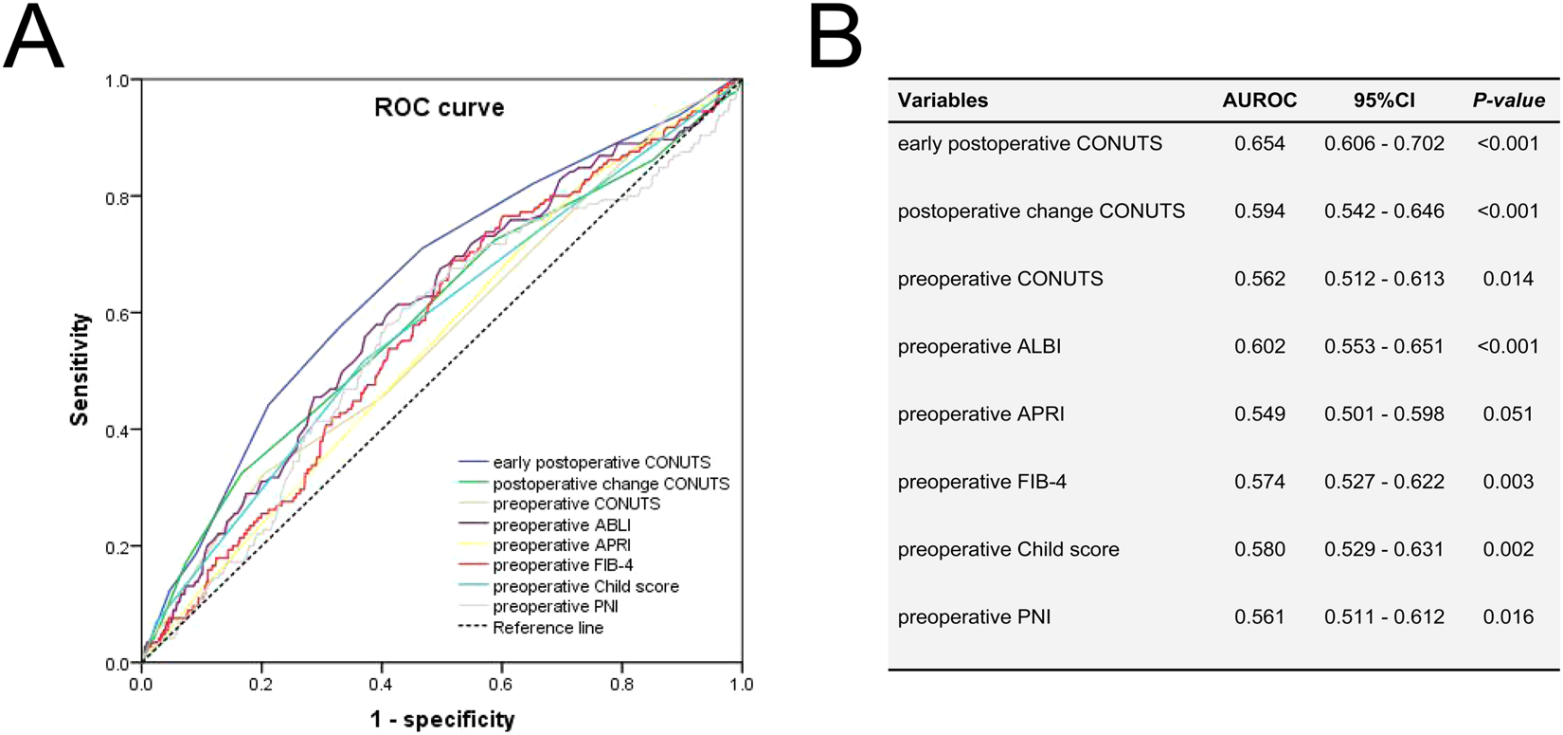
(**A**) The receiver operating characteristic curves for immune-nutritional indexes related to postoperative complication III-V. (**B**) The area under curves, standard error, *P*-value and 95% Confidence Interval of indexes in predicting complication III-V.

1334 consecutive patients were included in this study, including 1136 (85.2%) males and 198 (14.8%) females. 1208 (90.6%) patients with chronic HBV infection. Positive HBV-DNA load was detected in 436 (32.7%) patients. The median age of patients was 50 years old. Total tumor diameter was 7.3 ± 3.2 cm. The preoperative liver function of 1295 (97.1%) patients was classified as Child–Pugh A. 354 (26.5%) patients developed clinically relevant PHT. The other clinical parameters were shown in the Table 1. Several prognostic factors, including APRI, FIB-4, ALBI, PNI, Child score and CONUTS, were compared with ROC curve (Fig. 2A). The area under curves (AUCs), standard error, *P-*value and 95% Confidence Interval of factors in predicting complication III-V are shown in Fig. 2B. Early postoperative CONUTS (AUC = 0.654, *P* < 0.001) was selected as an optimal index and 8 was identified as the cut-off value. Then, patients were divided into the two groups: high CONUTS group (early postoperative CONUTS ≥ 8) with 659 patients and low CONUTS group (early postoperative CONUTS < 8) with 675 patients. There is no significant differences in HBV infection, HBV-DNA load, Child-Pugh grade, incidence of CSPH, platelet, tumor number, total tumor diameter, ASA grade, microvascular invasion, tumor differentiation and R-0 resection were observed between the two groups. Patients with early postoperative high CONUTS had more intraoperative blood loss (*P* < 0.001), transfusion rate (*P* < 0.001), occupancy rate of ICU (*P* < 0.001), longer hospitalization time (*P* < 0.001) and mortality in 90 days (*P* = 0.001). The details were shown in the Table 1.

**Table 1:** Patient characteristics and postoperative outcomes between patients with high- and low early postoperative CONUTS.

Parameters	All patients (n = 1334)	High CONUTS group (n = 659)	Low CONUTS group (n = 675)	*P-value*
		Early postoperative CONUTS ≥ 8	Early postoperative CONUTS < 8	
**Preoperative parameters**				
Male, n (%)	1136 (85.2%)	547 (83.0%)	589 (87.3%)	0.029
Age (years), median (IQR)	50 (41–60)	52 (43–60)	47 (40–58)	0.004
HBV-DNA >1000 U/mL, n (%)	436 (32.7%)	144 (33.7%)	244 (31.9%)	0.893
HBV infection (+), n (%)	1208 (90.6%)	609 (92.4%)	599 (88.7%)	0.054
Child Pugh grade A, n (%)	1295 (97.1%)	637 (96.7%)	658 (97.5%)	0.374
Lymphocytes (/mm^3^), median (IQR)	1.41 (1.10–1.73)	1.32 (1.05–1.67)	1.47 (1.17–1.79)	<0.001
Cholesterol (mg/dL), median (IQR)	4.09 (3.52–4.69)	3.97 (3.38–4.50)	4.19 (3.68–4.87)	<0.001
Total bilirubin (umol/L), median (IQR)	13.6 (10.4–18.6)	14.2 (10.7–18.8)	13.1 (10.2–18.3)	0.026
Albumin (g/L), median (IQR)	41.1 (38.2–43.8)	40.3 (37.2–43)	41.9 (39.2–44.6)	<0.001
INR, median (IQR)	1.3 (1.2–1.3)	1.3 (1.2–1.3)	1.3 (1.2–1.3)	0.039
Platelet (10^9^/L), median (IQR)	139 (97–191)	140 (96–202)	139 (99–182)	0.433
AST (U/L), median (IQR)	42 (30–61)	44 (32–67)	39 (29–53)	<0.001
ALT (U/L), median (IQR)	40 (29–59)	41 (29–62)	39 (28–57)	0.039
CSPH, n (%)	354 (26.5%)	179 (27.2%)	175 (25.9%)	0.609
Single tumor, n (%)	1172 (87.9%)	581 (88.2%)	591 (87.6%)	0.738
Total tumor diameter, n (%)	7.3 ± 3.2	7.4 ± 3.0	7.2 ± 3.4	0.305
**Intraoperative parameters**				
ASA grade III–IV, n (%)	310 (23.2%)	153 (23.2%)	157 (23.3%)	0.775
Hepatic inflow occlusion				0.096
Total, n (%)	174 (13.3%)	92 (14.0%)	82 (12.1%)	
Hemihepatic, n (%)	586 (43.9%)	270 (41.0%)	316 (46.8%)	
None, n (%)	574 (42.8%)	297 (45.1%)	277 (41.0%)	
Anatomic hepatectomy, n (%)	797 (59.7%)	426 (64.6%)	371 (55.0%)	<0.001
Major liver resection, n (%)	880 (66.0%)	448 (67.9%)	432 (64.0%)	0.124
**Operative procedure**				0.002
Extended left hemi-hepatectomy, n (%)	30 (2.2%)	21 (3.2%)	9 (1.3%)	
Extended right hemi-hepatectomy, n (%)	21 (1.6%)	9 (1.4%)	12 (1.8%)	
Left hemi-hepatectomy, n (%)	227 (17.0%)	112 (17.1%)	115 (16.9%)	
Right hemi-hepatectomy, n (%)	394 (29.5%)	221 (33.5%)	173 (25.6%)	
Wedge hepatectomy, n (%)	212 (15.9%)	92 (14.0%)	120 (17.8%)	
Segmental hepatectomy, n (%)	450 (33.7%)	204 (31.0%)	246 (36.4%)	
Blood loss (mL), median (IQR)	400 (250–600)	450 (300–800)	350 (200–500)	<0.001
Transfusion, n (%)	333 (25.0%)	213 (32.3%)	120 (17.8%)	<0.001
**Pathological results**				
Microvascular invasion, n (%)	215 (16.1%)	105 (15.9%)	110(16.3%)	0.882
Differentiation, n (%)				0.136
High	79 (5.9%)	43 (6.5%)	36 (5.3%)	
Moderate	1029 (77.1%)	493 (74.8%)	536 (79.4%)	
Low	226 (16.9%)	123 (15.3%)	103 (15.3%)	
Cirrhosis, n (%)	791 (59.3%)	424 (64.3%)	367 (54.4%)	<0.001
R0-resections, n (%)	1166 (87.4%)	565 (85.7%)	601 (89.0%)	0.083
**Postoperative parameters**				
Complication III-V, n (%)	145 (10.9%)	103 (15.6%)	42 (6.2%)	<0.001
ICU stay > 48 hours, n (%)	90 (6.7%)	62 (9.4%)	28 (4.1%)	<0.001
Postoperative hospitalization, (day), median (IQR)	10 (8–12)	10 (8–13)	9 (7–11)	<0.001
Mortality in 90 days, n (%)	20 (1.5%)	17 (2.6%)	3 (0.4%)	0.001

TBIL = total bilirubin, AST = aspartate aminotransferase, ALT = alanine aminotransferase, ALB = serum albumin, PLT = platelet.

INR = International Normalized Ratio, ASA = American Society of Anesthesiologists, CSPH = clinical significant portal hypertension.

MVI = micro-vascular invasion.

### Postoperative complications

A total of 444 patients suffered the postoperative complications, grade III-V complications appeared in 145 patients. 13 patients died of the severe complications, died causes were cardiovascular accident (1 case), secondary abdominal hemorrhage (2 cases), abdominal infection (3 case), liver failure grade C (3 cases), multiple organ dysfunction syndrome (4 cases). Patients with early postoperative high CONUTS had higher incidence of complication III-V (*P* < 0.001), pulmonary complications (*P* = 0.011), bile leakage (*P* = 0.018), intra-abdominal hemorrhage (*P* = 0.001), and liver failure Grade C (*P* = 0.002). The details were shown in the Table 2.

**Table 2:** postoperative complications between patients with high- and low early postoperative CONUTS.

Postoperative complications*	All patients(n = 1334)	High CONUTS group (n = 659)	Low CONUTS group (n = 675)	*P-value*
		Early postoperative CONUTS ≥ 8	Early postoperative CONUTS < 8	
Grade I, n (%)	120 (9.0%)	60 (9.1%)	60 (8.9%)	<0.001
Grade II, n (%)	179 (13.4%)	101 (15.3%)	78 (11.6%)	
Grade IIIa, n (%)	97 (7.3%)	64 (9.7%)	33 (4.9%)	
Grade IIIb, n (%)	5 (0.4%)	4 (0.6%)	1 (0.1%)	
Grade IVa, n (%)	28 (2.1%)	22 (3.3%)	6 (0.9%)	
Grade IVb, n (%)	2 (0.1%)	2 (0.3%)	0 (0.0%)	
Grade V, n (%)	13 (1.0%)	11 (1.7%)	2 (0.3%)	
Cardiovascular complications, n (%)	16 (1.2%)	8 (1.2%)	8 (1.2%)	0.962
Pulmonary complications, n (%)	132 (9.9%)	79 (12.0%)	53 (7.9%)	0.011
Neurological complication, n (%)	15 (1.1%)	9 (1.4%)	6 (0.9%)	0.409
Intracable ascites, n (%)	36 (2.7%)	18 (2.7%)	18 (2.7%)	0.942
Bile leakage, n (%)	23 (1.7%)	17 (2.6%)	6 (0.9%)	0.018
Intra-abdominal hemorrhage, n (%)	43 (3.2%)	32 (4.9%)	11 (1.6%)	0.001
#PLF A-B, n (%)	158 (11.8%)	65 (9.9%)	62 (9.2%)	0.826
#PLF C, n (%)	31 (2.3%)	24 (3.6%)	7 (1.0%)	0.002

^*^Postoperative complications were classified by Clavien-Dindo classification^21^.

^#^PLF = Posthepatectomy liver failure (ISGLS)^22^.

### Risk factors for postoperative complication III-V

In order to identify the risk factors for postoperative complication III-V, a univariate analysis was carried out. Preoperative cholesterol (*P* = 0.002), preoperative albumin (*P* < 0.001), Child-Pugh score (*P* < 0.001), early postoperative CONUTS ≥ 8 (*P* < 0.001), early postoperative albumin (*P* < 0.001), early postoperative cholesterol (*P* < 0.001), intraoperative blood loss (*P* < 0.001), transfusion (*P* < 0.001), major liver resection (*P* = 0.001), pathological indexes included tumor differentiation (*P* = 0.006) and cirrhosis (*P* = 0.039) were identified as the significant factors for postoperative complication III-V. The details were shown in the Table 3.

**Table 3:** Clinical parameters between patients with and without complication III-V after liver resection.

Parameters	Postoperative Complication III-V		*P*-value
	NO (n = 1189)	YES (n = 145)	
Male, n (%)	1009 (84.86%)	127 (87.58%)	0.384
Age (years), median (IQR)	50 (41–60)	52 (43–59)	0.577
HBV-DNA > 1000 U/mL, n (%)	386 (32.46%)	50 (34.48%)	0.625
HBV infection (+), n (%)	1071 (90.07%)	137 (94.48%)	0.175
Child Pugh score, mean (SD)	5.4 ± 0.5	5.6 ± 0.6	<0.001
Preoperative total lymphocytes (/mm^3^), median (IQR)	1.40 (1.10–1.73)	1.43 (1.12–1.74)	0.881
Preoperativel cholesterol (mg/dL), median (IQR)	4.11 (3.54–4.72)	3.89 (3.23–4.43	0.002
Preoperative albumin (g/L), median (IQR)	41.2 (38.3–43.9)	39.7 (36.8–42.8)	<0.001
Total bilirubin (umol/L), median (IQR)	13.6 (10.3–18.6)	13.9 (11.5–18.6)	0.285
INR, median (IQR)	1.3 (1.2–1.3)	1.3 (1.2–1.3)	0.707
Platelet (109/L), median (IQR)	140 (98–193)	128 (90–182)	0.106
AST (U/L), median (IQR)	41 (30–61)	45 (32–61)	0.054
ALT (U/L), median (IQR)	40 (28–59)	41 (30–60)	0.69
Early postoperative albumin (g/L), median (IQR)	30.4 (27.2–33.6)	27.7 (24.5–30.7)	<0.001
Early postoperative cholesterol (mg/dL), median (IQR)	254 (208–309)	202 (159–264)	<0.001
Early postoperative lymphocytes (/mm^3^), median (IQR)	820 (590–1130)	760 (560–1140)	0.257
Early postoperative CONUTS ≥ 8	556 (46.8%)	103 (71.0%)	<0.001
CSPH, n (%)	310 (26.1%)	44 (30.3%)	0.274
Single tumor, n (%)	1045 (87.9%)	127 (87.6%)	0.893
Total tumor diameter, n (%)	7.7 ± 3.3	7.2 ± 3.2	0.099
**Intraoperative parameters**			
ASA grade III–IV, n (%)	270 (22.70%)	40 (27.58%)	0.189
Anatomic hepatectomy, n (%)	719 (60.47%)	78 (53.79%)	0.122
Hepatic inflow occlusion			0.885
Total, n (%)	154 (12.95%)	20 (13.79%)	
Hemihepatic, n (%)	525 (44.15%)	61 (42.06%)	
None, n (%)	510 (42.89%)	64 (44.13%)	
**Operative procedure**			0.079
Extended left hemi-hepatectomy, n (%)	28 (2.4%)	2 (1.4%)	
Extended right hemi-hepatectomy, n (%)	15 (1.3%)	6 (4.1%)	
Left hemi-hepatectomy, n (%)	205 (17.2%)	22 (15.2%)	
Right hemi-hepatectomy, n (%)	355 (29.9%)	39 (26.9%)	
Wedge hepatectomy, n (%)	183 (15.4%)	29 (20.0%)	
Segmental hepatectomy, n (%)	403 (33.9%)	47 (32.4%)	
Anatomic hepatectomy, n (%)	719 (60.47%)	78 (53.79%)	0.122
Major liver resection, n (%)	767 (64.50%)	113 (77.93%)	0.001
Blood loss (mL), median (IQR)	400 (200–600)	500 (300–900)	<0.001
Transfusion, n (%)	255 (21.4%)	78 (53.8%)	<0.001
Pathological results			
Microvascular invasion, n (%)	192 (16.1%)	23 (15.9%)	0.93
Differentiation, n (%)			0.006
High	67 (5.6%)	12 (8.3%)	
Moderate	933 (78.5%)	96 (66.2%)	
Low	189 (15.9%)	37 (25.5%)	
Cirrhosis, n (%)	693 (58.3%)	98 (67.6%)	0.039
R0-resections, n (%)	1056 (88.8%)	131 (90.3%)	0.578

TBIL = total bilirubin, AST = aspartate aminotransferase, ALT = alanine aminotransferase, ALB = serum albumin, PLT = platelet.

INR = International Normalized Ratio, ASA = American Society of Anesthesiologists, CSPH = clinical significant portal hypertension.

MVI = micro-vascular invasion.

In order to control the potential confounding factors, multivariate logistic regression analysis was performed. Model 1, which included Child score, preoperative albumin, preoperative cholesterol, early postoperative CONUTS ≥ 8, blood loss, transfusion and major liver resection as independent variables, showed that early postoperative CONUTS ≥ 8 (OR = 2.054, 95% CI = 1.371–3.078, *P* < 0.001) and transfusion (OR = 3.235, 95% CI = 2.159–4.847, *P* < 0.001) were identified as the independent risk factors of postoperative complication III-V. Model 2, in which early postoperative CONUTS ≥ 8 was replaced with early postoperative albumin and cholesterol, demonstrated that transfusion (OR = 3.159, 95% CI = 2.054–4.859, *P* < 0.001), early postoperative albumin (OR = 1.054, 95% CI = 1.013–1.097, *P* = 0.009) and cholesterol (OR = 1.693, 95% CI = 1.265–2.264, *P* < 0.001) as the significant and independent factors associated with postoperative complication III-V. The details were shown in the Table 4.

**Table 4:** Multivariate analysis of independent risk factors for patient with complication III-V after liver resection.

Parameters	Model 1			Model 2		
	OR	95% CI	*P*-value	OR	95% CI	*P*-value
Child Pugh score	0.709	0.526–0.955	0.024	0.742	0.549–1.003	0.052
Preoperative albumin	1.005	0.965–1.047	0.809	1.008	0.967–1.051	0.692
Preoperative cholesterol	1.106	0.941–1.300	0.221	0.978	0.877–1.089	0.683
Early postoperative albumin				1.054	1.013–1.097	0.009
Early postoperative cholesterol				1.693	1.265–2.264	<0.001
Early postoperative CONUTS ≥ 8	2.054	1.371–3.078	<0.001			
Blood loss	1.000	1.000	0.395	1.000	1.000–1.001	0.421
Transfusion (Y)	3.235	2.159–4.847	<0.001	3.159	2.054–4.859	<0.001
Major liver resection (Y)	1.359	0.877–2.106	0.170	1.332	0.853–2.081	0.207
Cirrhosis (Y)	1.238	0.842–1.820	0.277	1.256	0.850–1.856	0.253

## Discussion

Severe postoperative complications would prolong the length of hospital stay, increase the morbidity and worsen the prognosis. The main complications after hepatectomy include pulmonary complication, neurological compilation, intractable ascites, bile leakage, intra-abdominal hemorrhage and liver failure. Nutritional intervention could improve the tolerance of patients for chemotherapy and surgery, decrease postoperative complications, and improve the prognosis^4,24,25^. Recently there’s increasing study^8–10,26,27^ focus on the nutritional status of patients and found that complication III-V after hepatectomy was not only associated with liver function reserve, but also with nutritional status.

The CONUT score is composed of serum albumin, cholesterol level and the lymphocyte count, which were associated with immune response, infection, inflammation, tissue repair and regeneration^26^. Hypoalbuminemia and hypocholesterolemia could predict postoperative complications and poor prognosis^9,28^. Lymphocyte is associated with immune response and tumor progression^29–31^. Previous studies^13,32^ indicated that HCC patients with preoperative high CONUTS had significantly lower recurrence-free survival (*P* = 0.011) and overall survival (*P* = 0.006) rates, however further analysis of severe postoperative complications were not carried out. Surgical trauma, blood loss and organic consumption may be closely correlated with postoperative hemodynamics and abnormal blood constituents, which may affect the predictive ability of preoperative CONUTS for postoperative outcomes. Complex liver structure combined with cirrhosis usually lead to more blood loss, then more fluid infusion or blood components transfusion were carried out. A series of prospective observational trials about evaluating the prognostic value of different nutritional scores in major abdominal surgery included hepatic surgery are ongoing. One study^33^, from the University of Heidelberg, has reported that none of the nutritional assessment scores before operation defined malnutrition relevant to complications after pancreatic surgery. Therefore, we hypothesized that early postoperative CONUTS may become a more meaningful risk factor for postoperative complication III-V than preoperative index.

In order to investigate the relationship between the early postoperative CONUTS and complications, we conducted a comparison of multiple ROC curves included Child score, APRI, FIB-4, ALBI and PNI. The results confirmed what we supposed, early postoperative CONUTS, not preoperative or postoperative change CONUTS, was chosen as an optimal index. The postoperative high CONUTS group had more intraoperative blood loss, transfusion, postoperative morbidity, mortality and longer hospital stay after operation. Univariate and multivariate analysis suggested that early postoperative CONUTS ≥ 8 (OR = 2.054, 95%CI = 1.371–3.078, *P* < 0.001) was identified as an independent risk factor for complication III-V. Once early postoperative high CONUTS appears, attention should be paid and took required measure.

Transfusion was identified as another independently risk factor for complication III-V in the study. Operative trauma, blood composition consumption and coagulation derangements are important factors, which were associated with transfusion. HCC patients with cirrhosis who usually had lower tolerance to general anesthesia and surgical trauma. Several studies reported that transfusion was an independent risk factor for postoperative complications after hepatectomy, which was consistent with our results. Intraoperative excessive bleeding would cause liver ischemia and worsen intestinal ischemia-reperfusion injury. The impairment of intestinal barrier function would lead to the bacterial translocation, increase the incidence of postoperative infections, prolong the time of hospitalization, and influence patients’prognosis^34,35^. The liver parenchyma bleeding, blood vessel injury and tumor hemorrhage are important factors leads to transfusion. Therefore, accurate operation, meticulous hemostasis, and shorten operation time could reduce unnecessary transfusion and postoperative complications. In addition, previous guidelines^36^ recommended that liver resection as an indication of albumin supplementation. Whether preventive albumin supplement could reduce postoperative complications remains controversial. However, it is recognized that component transfusion is beneficial to correct postoperative hypoalbuminemia, severe coagulation disorder and relieve refractory ascites.

The present study has some limitations. First, the single-center study could not reduce information bias and selection bias. Second, a retrospective study lacked the validation set and had the poor strength of argument. Third, influence of chemoembolization or anticancer drugs on peripheral lymphocyte count before liver resection cannot be ignored. At last, multi-center prospective studies are required to to evaluate the role of early postoperative CONUTS.

In conclusion, early postoperative CONUTS is a useful tool in assessing the postoperative nutritional status in HCC patients who underwent liver resection. Postoperative high CONUTS did not only influence postoperative complication III-V, but also increase the mortality. The predictive value should be further evaluated.

### Ethical review

This study was approved by the Clinical Research Ethics Committee of the West China Hospital, Sichuan University. Written informed consent was obtained from all patients according to the policies of the committee. All the methods used in this study were carried out according to the approved guidelines.
